# Cases of Acute Rheumatism Treated with Nitras Potassæ in Large Doses

**Published:** 1845-06-01

**Authors:** Alden S. Sprague


### Cases of Acute Rheumatism, treated with Nitras Potassæ, in large Doses.

Communicated for the Buffalo Medical Journal, by ALDEN S. SPRAGUE, M. D.

Below I give the results of the treatment of three cases of Rheumatism with large doses of Nitre.

I have not deemed it necessary to say anything relative to the pathology of the disease, nor, of the nature of the action of the remedy on the organism for its removal, but, merely, the facts as they occurred.

I am aware there is nothing new, or original, in the practice, yet it strongly corroborates that of several eminent Physicians.

The first case which I treated with Nitre in large doses, occurred in May, 1840. The patient, a gentleman of this city, aged about 35, of a bilious temperament, was attacked with the ordinary symptoms of fever—such as pain in the head, back, and limbs; chills succeeded by heat, nausea, furred tongue, restlessness, &c.

On seeing him, I gave him an emetic, which operated well, afterwards a cathartic of jalap and calomel, followed by treatment which produced free perspiration. Some of the important symptoms gave way; but he was now attacked with excessive pains in the joints, which became also, enormously swolen.

The pain and swelling attacked, successively, the upper, and the lower extremities, accompanied by a bounding pulse, and, in fact, all the appearances of acute rheumatism.

I now commenced giving him the following prescriptions; Sal Nitre, ℥ss Pulv. Dov. ℈ii mix; divide into eight powders and give one every three hours in any convenient vehicle;—warm fomentations to the affected parts.

This treatment was continued for twenty-four hours. Not perceiving any change in the case, one ounce of the salt, combined with the same quantity of Dovers powder as before given, was ordered every day for three days longer, when a decided amelioration of the condition of my patient was manifest. The dose was now diminished, and continued several days longer. The disease now assuming a more moderate character, the nitre was discontinued, and in about three weeks my patient was well.

In addition to the above, an occasional cathartic was given; with light diet and diluent drinks throughout.

Another case, more striking in its character, was treated by me in March, 1842. A young gentleman, aged about 20, was attacked with severe rheumatic fever. Its onset was sudden and terrific. The pain, and tumefaction attacked all his limbs simultaneously, accompanied with great heat, thirst, and bounding pulse. I commenced the treatment by giving calomel and jalap until it operated freely; with warm fermentations to the affected parts. Afterward I directed the following:—sal nitre, ℥i; pulv dov. ℈ii; to be divided into eight parts, and one to be given every three hours in thin gruel. The treatment was continued four days without mate rial change. The salt was now given at longer intervals, the patient convalesced rapidly, and was well in ten or twelve days.

Another case, manifesting some peculiarities, came under my care the present spring.

A young lady, aged 18, who had been subject to convulsions in her infancy, and to intestinal worms afterwards, was attacked with rheumatism, first in the knee and ankle of the right extremity, then in the upper extremity of the same side, followed by a similar affection of the opposite side. She had been treated with domestic remedies for three or four days, without any relief.

When I was called, she suffered dreadfully, and was entirely unable to help herself. Her stomach and bowels were immediately evacuated by a large dose of ipecac and calomel; and she was directed to take a drachm of Sal. Nitre, and three grains Pulv. Dov. every three hours, with warm fomentations to extremeties. At the end of three days, the disease began to abate, and by the tenth, every vestige of it had disappeared.

After the rheumatic fever had subsided, a slight attack of chorea manifested itself, which, however, soon subsided, on the administration of tonics and antispasmodics.

I have treated many other cases of rheumatic fever in such a manner, but the above are sufficient to subserve the objects I have in view in the present communication.

Buffalo, May 7th, 1845.

---

The Pathology and treatment of acute Rheumatism, are topics concerning which there is much diversity of opinion, and a notable absence of definite, rational principles. We should be glad to add to the above the results of the experience of other practical Physicians, with reference to the treatment. The reader will find several of the most prominent therapeutic methods now in vogue, presented and discussed in the April No. of the American Journal of Medical Sciences, (1845). He will also find in the ninth and tenth Nos. of Braithwaites Retrospect, the remedy which has been so useful in the hands of Dr. Sprague, warmly advocated by Dr. Bennet in a communication originally made to the London Lancet.—Editor.

